# Inhibitors of pan-PI3K Signaling Synergize with BRAF or MEK Inhibitors to Prevent *BRAF*-Mutant Melanoma Cell Growth

**DOI:** 10.3389/fonc.2015.00135

**Published:** 2015-06-16

**Authors:** Melanie Sweetlove, Emma Wrightson, Sharada Kolekar, Gordon W. Rewcastle, Bruce C. Baguley, Peter R. Shepherd, Stephen M. F. Jamieson

**Affiliations:** ^1^Auckland Cancer Society Research Centre, The University of Auckland, Auckland, New Zealand; ^2^Department of Molecular Medicine and Pathology, The University of Auckland, Auckland, New Zealand; ^3^Maurice Wilkins Centre for Molecular Biodiscovery, The University of Auckland, Auckland, New Zealand

**Keywords:** melanoma, BRAF, PI3K, mTOR, selumetinib, vemurafenib, BEZ235, ZSTK474

## Abstract

BRAF and MEK inhibitors have improved outcomes for patients with *BRAF*-mutant melanoma, but their efficacy is limited by both intrinsic and acquired resistances. Activation of the PI3K pathway can mediate resistance to these agents, providing a strong rationale for combination therapy in melanoma. Here, a panel of nine low-passage human metastatic melanoma cell lines with *BRAF* mutations was tested in cell proliferation and protein expression assays for sensitivity to inhibitors of MEK (selumetinib) and BRAF (vemurafenib) as single agents and in combination with inhibitors of pan-PI3K (ZSTK474), pan-PI3K/mTOR (BEZ235), individual PI3K isoforms (p110α, A66; p110β, TGX-221; p110γ, AS-252424; p110δ, idelalisib), or mTORC1/2 (KU-0063794). Selumetinib and vemurafenib potently inhibited cell proliferation in all cell lines, especially in those that expressed low levels of phosphorylated AKT (pAKT). ZSTK474 and BEZ235 also inhibited cell proliferation in all cell lines and enhanced the antitumor activity of selumetinib and vemurafenib in the majority of lines by either interacting synergistically or additively to increase potency or by inducing cytotoxicity by significantly increasing the magnitude of cell growth inhibition. Furthermore, ZSTK474 or BEZ235 combined with selumetinib to produce robust inhibition of pERK, pAKT, and pS6 expression and synergistic inhibition of NZM20 tumor growth. The inhibitors of individual PI3K isoforms or mTORC1/2 were less effective at inhibiting cell proliferation either as single agents or in combination with selumetinib or vemurafenib, although KU-0063794 synergistically interacted with vemurafenib and increased the magnitude of cell growth inhibition with selumetinib or vemurafenib in certain cell lines. Overall, these results suggest that the sensitivity of *BRAF*-mutant melanoma cells to BRAF or MEK inhibitors is at least partly mediated by activation of the PI3K pathway and can be enhanced by combined inhibition of the BRAF/MEK and PI3K/mTOR signaling pathways.

## Introduction

*BRAF* mutations leading to constitutive activation of the RAS/RAF/MEK/ERK pathway and increased cell cycle progression, differentiation, survival, migration, and angiogenesis are reported in 40–50% of melanoma cases (1). Therapeutic agents that selectively target BRAF (e.g., vemurafenib, dabrafenib) or its downstream substrate MEK (e.g., trametinib) can improve overall survival in *BRAF*-mutant metastatic melanoma patients (2–5); however, their use as monotherapy is limited by intrinsic and acquired resistance. While the majority (around 80%) of *BRAF*-mutant melanomas display some degree of tumor regression upon initial treatment with BRAF or MEK inhibitors, approximately 50% fail to meet threshold criteria for partial response and only 2–3% respond completely, implying a degree of intrinsic resistance in the majority of *BRAF*-mutant melanomas (2, 3, 6). Acquired resistance is also a major problem during treatment with BRAF or MEK inhibitors, with most patients demonstrating tumor progression within 5–7 months of the start of therapy (2, 7).

Among the multiple potential mechanisms of intrinsic and acquired resistance to BRAF and MEK inhibition that have been identified (8–14), the PI3K signaling pathway has been frequently implicated. Loss of functional PTEN occurs in 10–30% of melanomas, preventing negative regulation of PI3K activity, resulting in hyperactivation of AKT and, subsequently, in increased cell survival, proliferation, migration, and invasion (15). PTEN loss has been implicated in intrinsic resistance to both vemurafenib (16) and dabrafenib (17). Similarly, high expression of phosphorylated AKT (pAKT) appears to predict resistance to the MEK inhibitor selumetinib in melanoma patients (18) and to selumetinib and vemurafenib in cell lines (19–21). Reactivation of ERK signaling in the presence of inhibitor through mechanisms such as *MEK1* or *NRAS* mutation, dimeric RAF signaling, *BRAF* amplification, or COT upregulation (1, 8, 9, 11, 12) is the primary route for acquired resistance. Whole-exome sequencing has revealed that ERK reactivation mechanisms are present in 50–70% of tumors from drug-resistant patients, with multiple resistance mechanisms detected in some tumors (21, 22). Non-ERK-dependent acquired resistance can also arise through activation of the PI3K pathway by genetic alteration (21) or upregulation of growth factor receptors such as the platelet-derived growth factor receptor or the insulin-like growth factor receptor (19, 23, 24). Furthermore, persistent activity of mTORC1, which operates downstream of both the PI3K and RAS/RAF/MEK/ERK signaling pathways, can lead to resistance following BRAF or MEK inhibition (19, 25, 26). Conversely, compensatory signaling through the RAS/RAF/MEK/ERK pathway following receptor tyrosine kinase (RTK) upregulation may promote resistance to PI3K pathway inhibition (27–30).

Given the evidence indicating that the RAS/RAF/MEK/ERK and PI3K pathways co-operate in melanomagenesis, the extensive cross-talk that exists between the pathways (31), and the role of each pathway in resistance to inhibition of the other, a strong rationale exists for combined pathway inhibition in melanoma. In support of this, several early-phase clinical trials are currently underway for combined PI3K and BRAF/MEK inhibitors in melanoma, while preclinical melanoma models have reported synergistic growth inhibition and overcoming of acquired or intrinsic resistance to BRAF or MEK inhibitors with PI3K pathway inhibitors (19, 24, 32–35). However, few studies have assessed these combinations in the setting of intrinsic sensitivity to BRAF or MEK inhibitors in melanoma. Here, we selected a panel of low-passage *BRAF*-mutant melanoma cell lines that were established and maintained at 5% oxygen tension to mimic physiological oxygen concentrations and were sensitive to the MEK inhibitor selumetinib and the BRAF inhibitor vemurafenib. We evaluated the activity of individual or pan isoform inhibitors of PI3K and/or mTOR alone or in combination with selumetinib or vemurafenib in the cell line panel to investigate if inhibition of PI3K/mTOR signaling could enhance the antitumor activity of selumetinib or vemurafenib in *BRAF*-mutant melanoma.

## Materials and Methods

### Chemicals

A66 (36), ZSTK474 (37), BEZ235 (38), TGX-221, and AS-252424 (39) were synthesized at the Auckland Cancer Society Research Centre as previously described. Selumetinib (Selleck Chemicals and LC Laboratories), vemurafenib (Medkoo Biosciences), idelalisib (Symansis), and KU-0063794 (Selleck Chemicals) were supplied as indicated.

BEZ235 was prepared as a dimethanesulfonate salt by treating a suspension of the solid in methanol with 2.2 equivalents of methanesulfonic acid, to give a clear solution. Dilution with ethyl acetate gave a precipitate, which was collected by filtration and washed with further ethyl acetate. Recrystallization from methanol–ethyl acetate gave the dimethanesulfonate salt as a pale yellow solid: mp 283–286°C; ^1^H NMR (400 MHz, DMSO-*d_6_*) δ 9.49 (s, 1H), 8.86 (d, *J* = 2.3 Hz, 1H), 8.52 (d, *J* = 2.1 Hz, 1H), 8.47 (dd, *J* = 9.0, 1.9 Hz, 1H), 8.39 (d, *J* = 9.0 Hz, 1H), 8.06–8.09 (m, 2H), 7.95–7.99 (m, 2H), 7.85–7.90 (m, 3H), 7.73 (ddd, *J* = 8.0, 7.0, 1.0 Hz, 1H), 7.37 (d, *J* = 1.7 Hz, 1H), 4.85 (br, exchangeable with D_2_O, 2H), 3.70 (s, 3H), 2.39 (s, 6H), 1.79 (s, 6H); Combustion Analysis, Calc. for C_32_H_31_N_5_O_7_S_2_: C, 58.1; H, 4.7; N, 10.6. Found: C, 58.0; H, 4.6; N, 10.65%.

### Cell culture

A panel of nine melanoma cell lines was chosen from a series of lines generated from surgical samples of metastatic melanoma obtained with appropriate consent from patients throughout New Zealand as previously described (40). The cell lines were maintained in α-modified minimal essential medium (Life Technologies) supplemented with insulin (5 μg/ml), transferrin (5 μg/ml), and sodium selenite (5 ng/ml; Roche Applied Sciences), 100 U/ml of penicillin, 100 μg/ml of streptomycin (Life Technologies), and 5% fetal calf serum (Moregate Biotech). The cell lines were cultured under low-oxygen conditions (5% O_2_, 5% CO_2_) at 37°C.

### Gene mutation profiling

The mutation status of 32 common driver oncogenes and h*TERT* was determined in the melanoma cell lines by Sequenom analysis. DNA was extracted using PureLinkTM Genomic DNA kit (Life Technologies), according to manufacturer’s protocol. To remove the EDTA-based elution buffer, DNA was re-precipitated into milliQ water. This was achieved by addition of ethanol and 5M ammonium acetate at −80°C for 2 h and centrifugation at 18,000 × *g* for 30 min at 4°C. The pellet was resuspended in ethanol and re-centrifuged at 18,000 × *g* for 10 min at 4°C, prior to resuspension in milliQ water. Extracted DNA was evaluated for gene mutations on the Sequenom MassARRAY^®^ using the MassARRAY OncoCartaTM Panel v 1.0 and the MelaCartaTM Panel v1.0 plus *hTERT*, according to manufacturer’s protocol. Analysis was carried out using Sequenom MassARRAY Typer Analyzer 4.0 genotyping software. *PTEN* mutation status was determined by PCR sequencing as described previously (41).

### Cell proliferation

Cells were seeded into 96-well plates at 10,000 cells per well and left to settle for 24 h at 37°C with 5% CO_2_ and 5% O_2_. Compounds were added to each plate at a range of concentrations in 0.2% or less DMSO. For combination studies, both compounds were tested at equivalent concentrations. Plates were returned to the incubator for 72 h before fixing in 10% trichloroacetic acid at 4°C for 1 h and staining with 0.4% sulforhodamine B (Sigma-Aldrich) in 1% acetic acid for 30 min in the dark at room temperature. Plates were washed in 1% acetic acid, dried, and incubated with unbuffered Tris base (10 mM; Serva) for 30 min on a plate shaker in the dark to solubilize the stain. The plates were read on a BioTek EL808 microplate reader at an absorbance of 490 nm with a reference wavelength of 450 nm. Absorbances of treated cells were compared to untreated cells at 0 h (100% growth inhibition) and 72 h (0% growth inhibition) after treatment. Growth inhibition above 100% indicated that fewer cells were present than when the compounds were first administered. EC_50_ values were calculated by fitting the inhibition data to a four-parameter logistic sigmoidal dose–response curve using GraphPad Prism 6.01. Combination indices (CI) were calculated at EC_50_ using the method of Chou and Talalay (42). CI values <0.7 indicated synergy, 0.7–0.9 indicated weak synergy, 0.9–1.1 indicated additivity, 1.1–1.45 indicated weak antagonism, and >1.45 indicated antagonism.

### Western blotting

Untreated cells for basal protein expression or cells treated with 500 nM of compound for 1 or 24 h were lysed in lysis buffer containing 1% protease inhibitor cocktail (Sigma-Aldrich) on ice for 15–30 min. Cells were centrifuged at 13,000 rpm for 10 min at 4°C to remove insoluble material. Protein concentration of cell lysates was determined by bicinchoninic acid assay (Thermo Scientific) against bovine serum albumin (BSA; Immuno-Chemical Products Ltd.) standards at an absorbance of 562 nm on a BioTek Synergy HT plate reader using KC4 v3.4 software. Forty micrograms of each lysate was loaded onto polyacrylamide gels (10% acrylamide) and separated by SDS-PAGE at 120V for 90 min. Each gel was transferred onto an Immobilin^®^ PVDF membrane (Sigma-Aldrich) at 25V for 12 min on a BioRad Trans-Blot^®^ TurboTM semi-dry transfer machine. Following protein transfer, membranes were incubated in blocking buffer [Tris-buffered saline with 0.5% Tween^®^-20 (Serva) and 3% BSA] for at least 30 min then cut and incubated overnight at 4°C with antibodies at 1:1000 dilution (unless indicated) against either pAKT (Ser473, Thr308), pERK1/2 (Thr202/Tyr204), pS6 (Ser235/Ser236, 1:2000; Ser240/Ser244, 1:2000), AKT, ERK1/2, S6 (1:2000), PTEN (1:100), IGF1Rβ, EGFR, Insulin Receptor β, c-MET, ERBB3, MERTK (all Cell Signaling Technologies), and β-actin (1:2000; Sigma-Aldrich). Membranes were washed then incubated with anti-mouse (1:20,000; Sigma-Aldrich) or anti-rabbit (1:4000–5000; Dako) goat IgG HRP-conjugated secondary antibody in blocking buffer for 1 h at room temperature. After further washes, the membranes were incubated with BioRad ClarityTM ECL or Perkin Elmer Western Lighting Ultra (pAKT membranes) for 4 min prior to imaging on a LAS-4000 luminescent image analyzer (Fujifilm). After visualization of phosphorylated proteins, membranes were stripped and reprobed with total proteins and β-actin. β-actin was used to confirm equal protein loading in each blot. Each cell lysate was tested in duplicate in two to three independent experiments.

### Tumor growth inhibition in NZM20 xenograft model

Age-matched specific pathogen-free female NIH-III mice were subcutaneously inoculated with 5 × 10^6^ NZM20 cells. Treatment was initiated when tumors reached 150 mm^3^ in volume as measured by electronic calipers. The dosing vehicles used were 0.5% hydroxypropyl methylcellulose with 0.2% Tween 80 (selumetinib and control vehicle), 2% carboxymethylcellulose with 1% Tween 80 (ZSTK474), and 40% PEG-400 in 20% hydroxypropyl-β-cyclodextrin (BEZ235). All vehicle constituents were supplied by Sigma-Aldrich. Treatments were administered daily for 14 days at half the single agent maximum tolerated dose as the free base equivalent by oral gavage at a volume of 10 ml/kg. Animals were monitored daily for bodyweight and any observational signs of toxicity. Tumor growth inhibition (TGI) was calculated by determining the tumor size of drug-treated mice relative to starting size as a percentage of the average relative tumor size of control mice. Synergy was inferred if the TGI of the combination was greater than the product of the TGI values of each single agent. All animal experiments followed protocols approved by the University of Auckland Animal Ethics Committee.

## Results

### Mutation status and protein expression of *BRAF*-mutant melanoma cell line panel

We selected a panel of nine early passage *BRAF*-mutant melanoma cell lines that were both developed and maintained at low-oxygen tension (5%). The mutation status of common oncogenic driver mutations in these lines was determined by Sequenom or PCR profiling. Seven of the cell lines contained V600E mutations in *BRAF*, while two contained V600K mutations (Table 1). Three lines contained confirmed frameshift mutations or deletions in *PTEN* and five lines had C250T or C228T mutations in *hTERT*. No mutations were detected in *ABL1*, *AKT1*, *AKT2*, *AKT3*, *CDK4*, *CTNNB1*, *CXCR4*, *EGFR*, *EPHA10*, *EPHB6*, *ERBB2*, *ERBB4*, *FGFR1*, *FGFR3*, *FLT3*, *GNA11*, *GNAQ*, *HRAS*, *JAK2*, *KIT*, *KRAS*, *MEK*, *MET*, *NEK10*, *NRAS*, *PDGFA*, *PDGFRA*, *PIK3CA*, *PTK2B*, *RET*, and *ROR2*.

**Table 1:** **Gene mutation status of *BRAF*, *PIK3CA*, *PTEN*, and *hTERT* and protein expression of PTEN and pAKT in the melanoma cell line panel**.

Cell line	Mutation status				Protein expression	
	*BRAF*	*PIK3CA*	*PTEN*	*hTERT*	PTEN	pAKT
NZM3	V600K	WT	WT	WT	+++	+/−
NZM6	V600E	WT	Exon 3 deletion	WT	−	+++
NZM7	V600E	WT	WT	C250T	+++	++
NZM11	V600E	WT	WT	WT	+++	+/−
NZM12	V600E	WT	WT	C250T	+++	++
NZM20	V600E	WT	WT	C228T	+++	+/−
NZM30	V600E	WT	WT (exons 2–9) Exon 1 unknown	C228T	−	+++
NZM34	V600E	WT	Exon 5 frameshift	C250T	−	++
NZM43	V600K	WT	Exon 1 frameshift	WT	−	++

*WT, wild type; +++, high expression; ++, moderate expression; +, low expression; +/−, not expressed in some blots and low expression in others; −, not expressed in all blots*.

**Figure 1:**
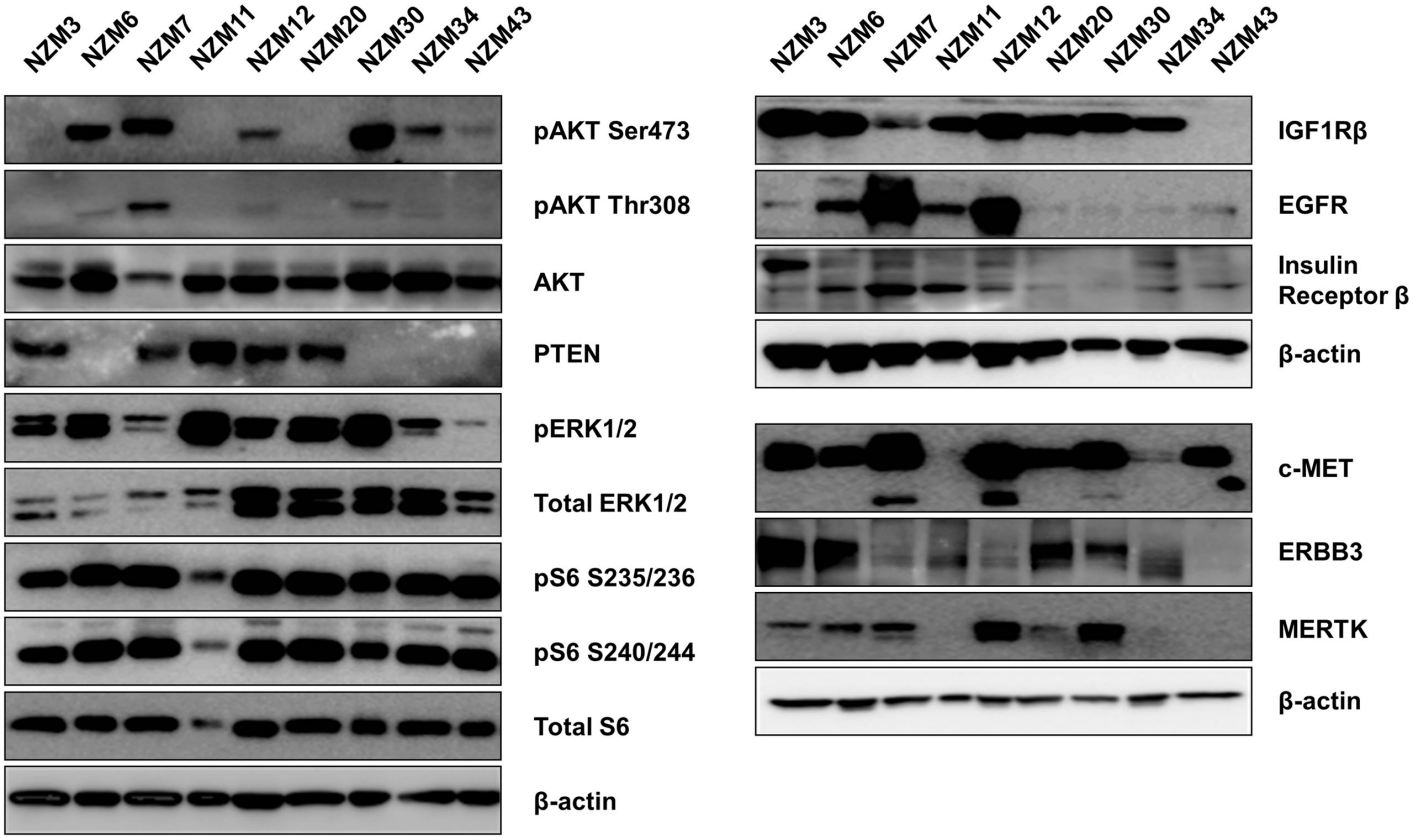
**Basal expression of key signaling molecules and receptor tyrosine kinases involved in the PI3K/mTOR and RAS/RAF/MEK/ERK signaling pathways**. Each blot is a representative image of two to three independent cell lysates.

The basal protein expression of phosphorylated and/or total forms of AKT, PTEN, ERK1/2, and S6 were determined in the cell line panel by western blotting (Figure 1). Minor differences in basal expression were detected between different lysates of the same cell line (e.g., compare Figure 1 to control lysates in Figures 5 and 6), but generally the same pattern of expression was evident. pAKT was variably expressed across the cell line panel with similar expression at ser473 and thr308 phosphorylation sites. Cell lines with low to moderate pAKT expression had high-PTEN expression (NZM3, NZM11, NZM12, and NZM20), unless they were *PTEN*-mutant (NZM34, NZM43). All *PTEN*-mutant lines, along with NZM30, lacked PTEN expression. pERK was expressed in all cell lines at variable levels, with the lowest expression detected in NZM7 and NZM43, while pS6 was expressed in all cell lines at similar levels relative to total protein.

Several RTKs that can activate PI3K/mTOR or RAS/RAF/MEK/ERK signaling pathways were expressed in the NZM lines (Figure 1). High-EGFR expression was observed in NZM7 and NZM12, which may have accounted for the moderate to high pAKT expression in these two lines, despite the expression of PTEN. The low expression of RTKs (apart from c-MET) in NZM43 may explain why pERK and pAKT signaling was relatively low in this line compared to the other *BRAF*-mutant and PTEN-null cell lines.

### *BRAF*-mutant melanoma lines with low-pAKT expression are more sensitive to selumetinib and vemurafenib

**Figure 2:**
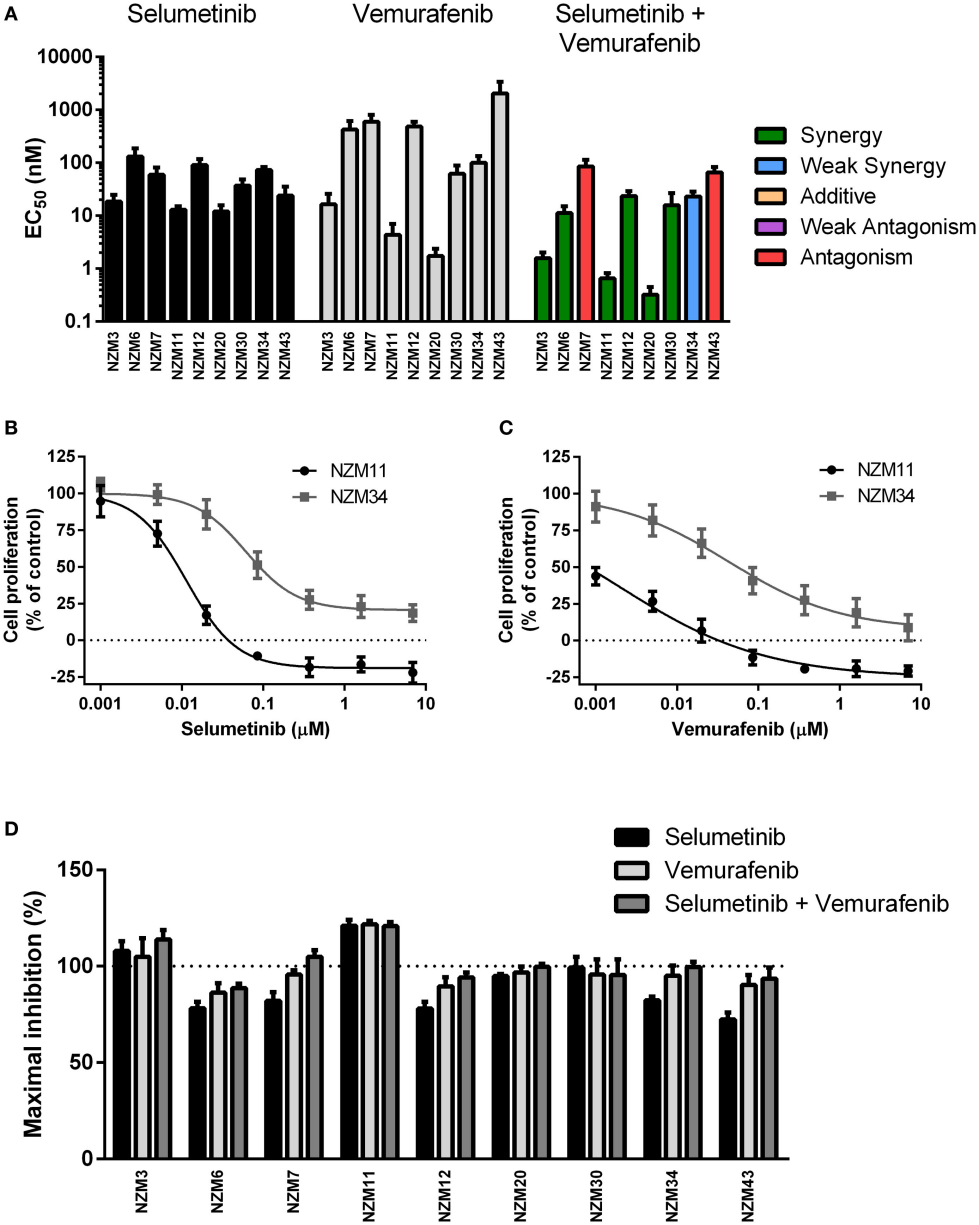
**Selumetinib and vemurafenib potently inhibit cell proliferation in *BRAF*-mutant melanoma cell lines**. **(A)** EC_50_ values for selumetinib, vemurafenib, and selumetinib + vemurafenib with combination interactions shown based on the CI values at EC_50_. **(B)** Growth profile for NZM11 and NZM34 cells treated with selumetinib or **(C)** vemurafenib. **(D)** Maximum inhibition of cell proliferation relative to pretreatment in NZM cell lines after 72 h exposure to selumetinib, vemurafenib or selumetinib, and vemurafenib at concentrations up to 10 μM. Bars or symbols represent the mean ± SEM of *n* = 3–6. CI values <0.7 indicated synergy, 0.7–0.9 indicated weak synergy, 0.9–1.1 indicated additivity, 1.1–1.45 indicated weak antagonism, and >1.45 indicated antagonism.

Each cell line in the panel was exposed to multiple concentrations of selumetinib or vemurafenib to determine the effects of these drugs on cell proliferation. Selumetinib was highly effective at inhibiting cell proliferation in all cell lines, with EC_50_’s ranging from 12.0 ± 3.7 to 131 ± 56 nM (Figure 2A). The lowest EC_50_ values were observed in low-pAKT-expressing cell lines NZM3, NZM11, NZM20, and NZM43. Similarly, vemurafenib effectively inhibited cell proliferation in all cell lines, generating EC_50_ values <20 nM in NZM3, NZM11, and NZM20. However, vemurafenib was almost 100-fold less potent than selumetinib in NZM43 cells (EC_50_ = 2.1 ± 1.2 μM vs. 24.2 ± 10.1 nM). There was no clear relationship between basal pERK protein expression and selumetinib or vemurafenib sensitivity in the cell line panel.

Since BRAF and MEK inhibitors have recently shown promise as combination therapy for *BRAF*-mutant melanoma (5), the effectiveness of selumetinib and vemurafenib in combination at preventing cell proliferation was also determined. All cell lines, except NZM7 and NZM43, the two with lowest basal pERK expression, showed synergistic interactions at EC_50_ when the drugs were administered in combination (Figure 2A; Table 2). On examining the EC_50_ profiles, it became apparent that there was variability among the cell lines in the extent of proliferation that could be inhibited by selumetinib (Figure 2B) and vemurafenib (Figure 2C). In most cell lines, selumetinib and to a lesser extent vemurafenib at concentrations up to 10 μM were unable to inhibit cell proliferation by 100% back to pretreatment cell counts (Figure 2D). The major exceptions were low-pAKT-expressing NZM3 and NZM11, where both selumetinib and vemurafenib inhibited cell proliferation by >110 and >120%, respectively, indicating that the drugs had a cytotoxic response on these cell lines. Combining selumetinib and vemurafenib offered no significant increase in maximum inhibition in cell proliferation compared to both agents administered alone in any cell line, such that inhibition of proliferation failed to reach 100% in six of the nine cell lines.

**Table 2:** **Combination indices at EC_50_ for the combination treatment groups relative to each single agent**.

Cell line	Selumetinib + Vemurafenib	Selumetinib + ZSTK474	Selumetinib + BEZ235	Vemurafenib + ZSTK474	Vemurafenib + BEZ235
NZM3	0.35 ± 0.07	0.92 ± 0.05	0.87 ± 0.12	0.92 ± 0.26	0.72 ± 0.25
NZM6	0.32 ± 0.09	0.91 ± 0.05	0.97 ± 0.05	0.46 ± 0.05	0.73 ± 0.17
NZM7	3.30 ± 1.23	2.47 ± 0.86	1.36 ± 0.38	0.24 ± 0.03	0.31 ± 0.03
NZM11	0.45 ± 0.05	0.81 ± 0.21	1.47 ± 0.35	1.58 ± 0.58	1.29 ± 0.20
NZM12	0.68 ± 0.27	0.91 ± 0.21	1.08 ± 0.09	0.36 ± 0.02	0.87 ± 0.12
NZM20	0.32 ± 0.07	0.99 ± 0.12	1.07 ± 0.07	1.02 ± 0.73	1.11 ± 0.27
NZM30	0.69 ± 0.43	1.38 ± 0.27	0.87 ± 0.04	0.65 ± 0.23	0.68 ± 0.05
NZM34	0.78 ± 0.14	1.28 ± 0.11	1.07 ± 0.11	0.60 ± 0.11	0.62 ± 0.15
NZM43	5.98 ± 2.53	1.89 ± 0.22	1.33 ± 0.32	0.37 ± 0.09	2.86 ± 1.13

*Data are mean ± SEM (*n* = 3–5)*.

### ZSTK474 and BEZ235 can enhance the antiproliferative activity of selumetinib or vemurafenib in *BRAF*-mutant melanoma cell lines

**Figure 3:**
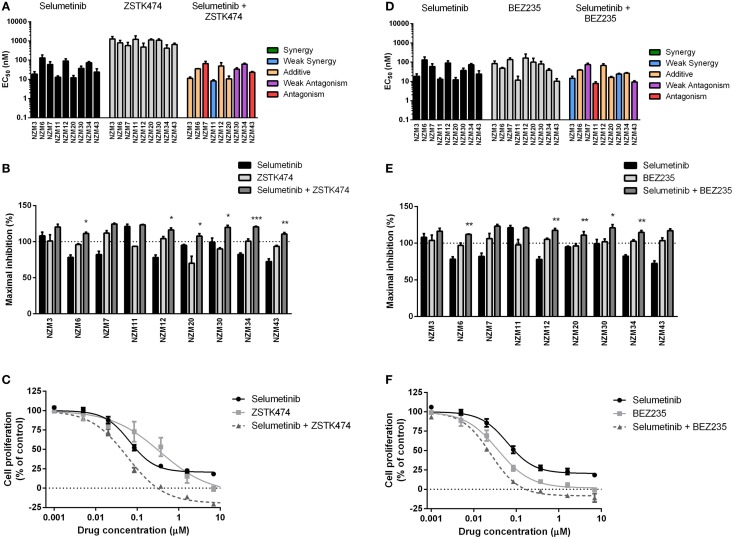
**Combining ZSTK474 or BEZ235 with selumetinib has little effect on potency but increases maximal inhibition of cell proliferation**. **(A)** EC_50_ values for selumetinib, ZSTK474, and selumetinib + ZSTK474 with combination interactions shown based on the CI values at EC_50_. **(B)** Maximum inhibition of cell proliferation relative to pretreatment after 72 h exposure to selumetinib, ZSTK474, or selumetinib + ZSTK474 at concentrations up to 10 μM. **(C)** Growth profile for NZM34 cells treated with selumetinib, ZSTK474, or selumetinib + ZSTK474. **(D–F)** As for **(A–C)** but with BEZ235 replacing ZSTK474. Bars or symbols represent the mean ± SEM of *n* = 3–7. Statistical significance of differences between mean values was evaluated by one-way ANOVA with Dunnett’s multiple comparison analysis. *P < 0.05; **P < 0.01; ***P < 0.001 relative to both single agent treatments. CI values <0.7 indicated synergy, 0.7–0.9 indicated weak synergy, 0.9–1.1 indicated additivity, 1.1–1.45 indicated weak antagonism, and >1.45 indicated antagonism.

Since the antiproliferative activity of selumetinib and vemurafenib was less pronounced in *BRAF*-mutant melanoma cell lines that expressed pAKT, we investigated if inhibition of PI3K activity could promote growth inhibition in *BRAF*-mutant melanoma cell lines. To achieve this, the pan-PI3K inhibitor ZSTK474 and the dual PI3K/mTOR inhibitor BEZ235 were administered to the cell line panel either alone or in combination with selumetinib or vemurafenib. ZSTK474 effectively inhibited cell proliferation across the cell line panel with EC_50_’s ranging from 416 ± 211 to 1278 ± 390 nM (Figure 3A); however, maximal inhibition only reached 100% in four out of nine cell lines at concentrations up to 10 μM (Figure 3B), indicating that ZSTK474 was unable to elicit a full cytostatic response in the majority of the NZM lines. Despite only modest synergy or additivity at EC_50_ in five lines when selumetinib and ZSTK474 were administered in combination (Figure 3A; Table 2), maximal inhibition was significantly increased relative to both single agents in all NZM lines (*P* < 0.05) except NZM3, NZM7, and NZM11, where selumetinib or ZSTK474 alone inhibited growth by >105% (Figure 3B). As a result, growth inhibition exceeded 100% in all lines treated with selumetinib and ZSTK474 in combination, indicating a cytotoxic response. This response is evident in the growth curves for these agents in NZM34 (Figure 3C), where a weak antagonistic interaction was observed for the combination at EC_50_, yet maximum inhibition was significantly increased from 82.3 ± 2.1 or 100.7 ± 3.0% with selumetinib or ZSTK474 alone to 120.5 ± 0.8% with the combination (*P* < 0.001 vs. both single agents).

BEZ235 was highly potent across the cell line panel with EC_50_’s ranging from 10.2 ± 2.8 to 163 ± 100 nM (Figure 3D) and was able to inhibit cell proliferation to a greater extent than either ZSTK474 or selumetinib with >100% inhibition observed in seven of nine cell lines (Figure 3E). Similarly to the combination of ZSTK474 with selumetinib, combining BEZ235 with selumetinib did not induce a synergistic interaction in the majority of cell lines, although maximal inhibition was significantly increased relative to both single agents in five cell lines (*P* < 0.05), as shown for NZM34 (Figure 3F), such that maximal growth inhibition exceeded 100% in all cell lines treated with BEZ235 and selumetinib. The only cell lines that did not show a >10% increase in maximal growth inhibition with combination treatment relative to single agent treatment were NZM3 and NZM11, where growth inhibition exceeded 100% with selumetinib alone.

**Figure 4:**
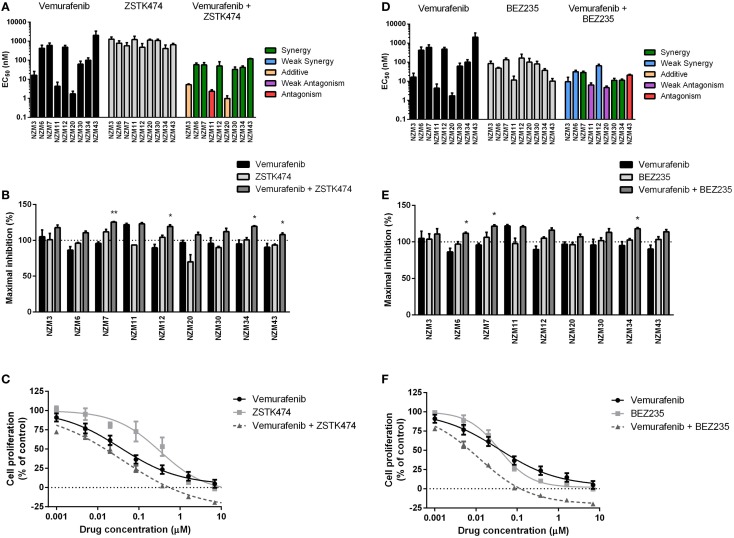
**Vemurafenib interacts with ZSTK474 or BEZ235 for enhanced antiproliferative activity**. **(A)** EC_50_ values for vemurafenib, ZSTK474, and vemurafenib + ZSTK474 with combination interactions shown based on the CI values at EC_50_. **(B)** Maximum inhibition of cell proliferation relative to pretreatment after 72 h exposure to vemurafenib, ZSTK474, or vemurafenib + ZSTK474 at concentrations up to 10 μM. **(C)** Growth profile for NZM34 cells treated with vemurafenib, ZSTK474, or vemurafenib + ZSTK474. **(D–F)** As for **(A–C)** but with BEZ235 replacing ZSTK474. Bars or symbols represent the mean ± SEM of *n* = 3–7. Statistical significance of differences between mean values was evaluated by one-way ANOVA with Dunnett’s multiple comparison analysis. *P < 0.05; **P < 0.01 relative to both single agent treatments. CI values <0.7 indicated synergy, 0.7–0.9 indicated weak synergy, 0.9–1.1 indicated additivity, 1.1–1.45 indicated weak antagonism, and >1.45 indicated antagonism.

For vemurafenib, synergy (CI < 0.7) was observed with ZSTK474 in the six cell lines that were least sensitive to vemurafenib as a single agent (Figure 4A; Table 2), but significant changes in maximal inhibition were observed in only NZM7, NZM12, NZM34, and NZM43 (*P* < 0.05) (Figures 4B,C). Likewise, vemurafenib and BEZ235 had a synergistic or weakly synergistic interaction in six cell lines, with the exceptions being NZM11, NZM20, and NZM43, which were highly sensitive (EC_50_ ≤ 10 nM) to either vemurafenib or BEZ235 as single agents (Figure 4D). Significant changes in maximum inhibition with this combination were limited to NZM6, NZM7, and NZM34 (*P* < 0.05) (Figures 4E,F). Both vemurafenib combinations were able to inhibit cell proliferation by >100% in all cell lines.

### Selumetinib in combination with ZSTK474 or BEZ235 inhibits pERK, pAKT, and pS6 expression

**Figure 5:**
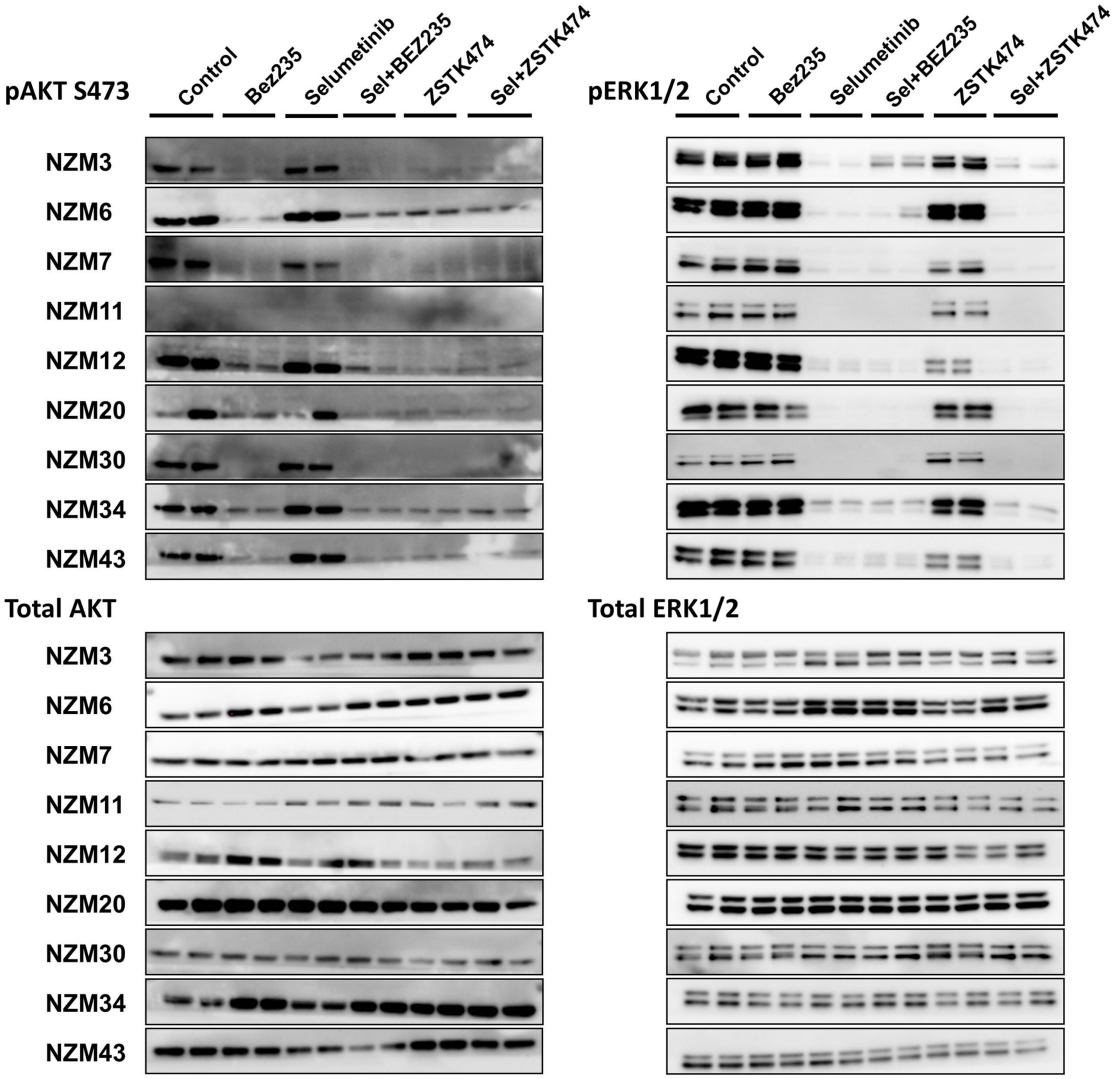
**Selumetinib (Sel) in combination with BEZ235 or ZSTK474 inhibits pAKT and pERK expression in *BRAF*-mutant melanoma cell lines**. Cells were treated with 500 nM of each compound as indicated for 1 h. Blots are representative images of two independent determinations.

To determine if selumetinib in combination with ZSTK474 or BEZ235 can prevent both ERK and AKT signaling, pERK and pAKT expression levels were determined by western blotting 1 and 24 h after 500 nM drug treatment (Figure 5). pAKT was investigated only at the ser473 phosphorylation site, since the basal expression at thr308 was too weak (Figure 2) to evaluate drug activity across the cell line panel. Treatment with selumetinib alone had no effect on pAKT expression, but was able to effectively inhibit pERK expression in all nine cell lines. By contrast, ZSTK474 and BEZ235 both inhibited pAKT expression but were largely ineffective at inhibiting pERK. In combination, selumetinib with either ZSTK474 or BEZ235 inhibited both pAKT and pERK expressions in all cell lines. A similar extent of pAKT and pERK inhibition was present after 1 or 24 h incubation (data not shown) with all treatments in all cell lines tested.

**Figure 6:**
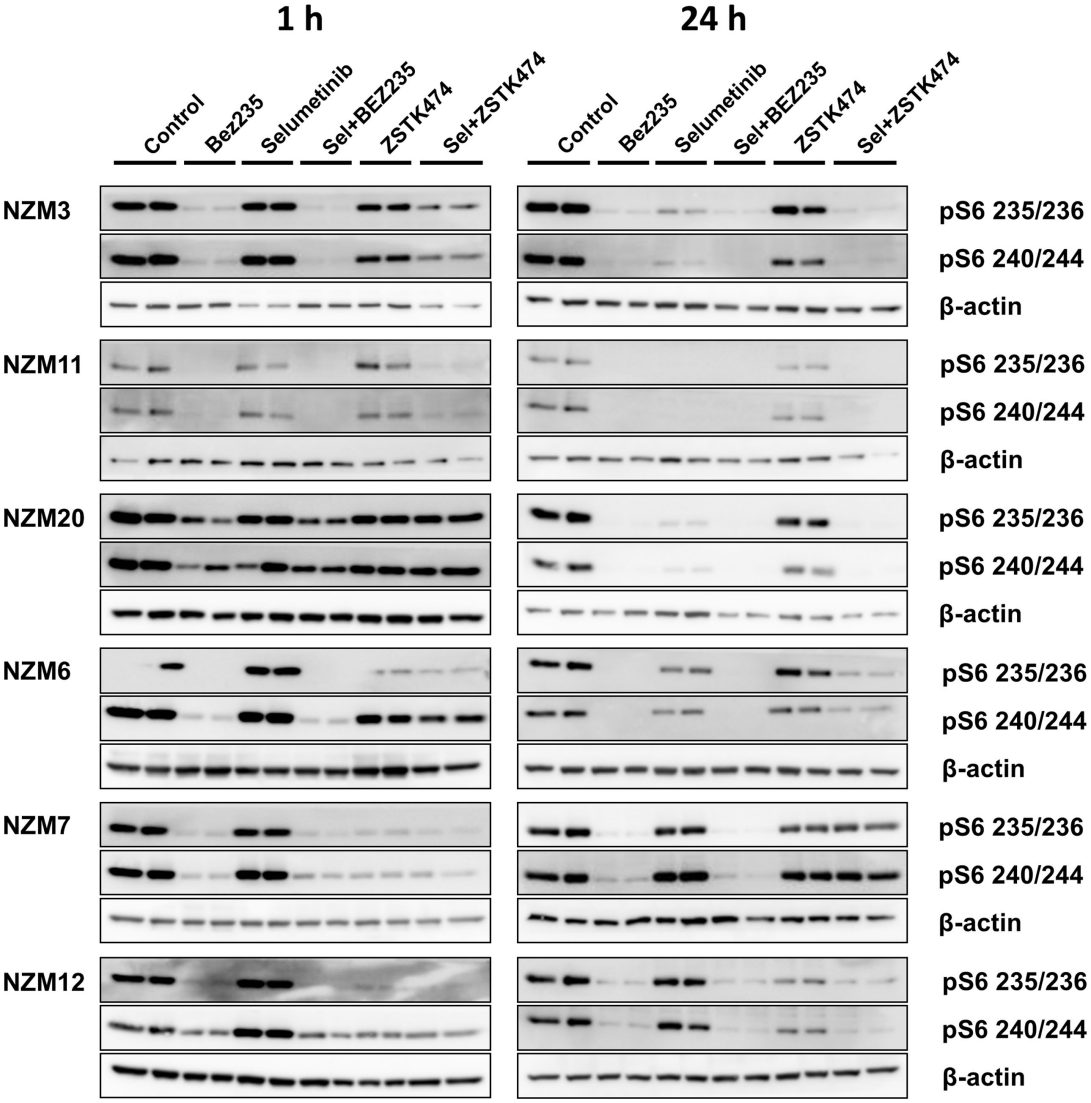
**Selumetinib (Sel) in combination with BEZ235 or ZSTK474 inhibits pS6 expression in *BRAF*-mutant melanoma cell lines that are highly sensitive (NZM3, NZM11, NZM20) or moderately sensitive (NZM6, NZM7, NZM12) to single agent selumetinib**. Cells were treated with 500 nM of each compound as indicated for 1 or 24 h. Blots are representative images of two independent determinations.

Since pS6 expression can predict responsiveness to BRAF and MEK inhibitors in *BRAF*-mutant melanoma cells (25, 26), we also investigated pS6 expression after 1 or 24 h treatment with selumetinib, ZSTK474 and BEZ235 alone and in combination with three cell lines that were highly sensitive to selumetinib and vemurafenib (NZM3, NZM11, and NZM20) and three cell lines that were less sensitive (NZM6, NZM7, and NZM12). Selumetinib had little effect on pS6 1 h after treatment in all cell lines, but after 24 h treatment caused greater inhibition of pS6 in the three highly sensitive lines (Figure 6). ZSTK474 inhibited pS6 after 1 h in NZM7 and NZM12, but this partially recovered with 24 h treatment. ZSTK474 was ineffective in the other cell lines. BEZ235 inhibited pS6 in all cell lines at 1 and 24 h as did the combination of BEZ235 with selumetinib. ZSTK474 combined with selumetinib inhibited pS6 at a greater extent than either single agent alone in all cell lines at either 1 or 24 h treatment, except NZM7.

### Inhibitors of individual PI3K isoforms or mTOR can interact with selumetinib or vemurafenib to induce increased inhibition of cell proliferation

**Figure 7:**
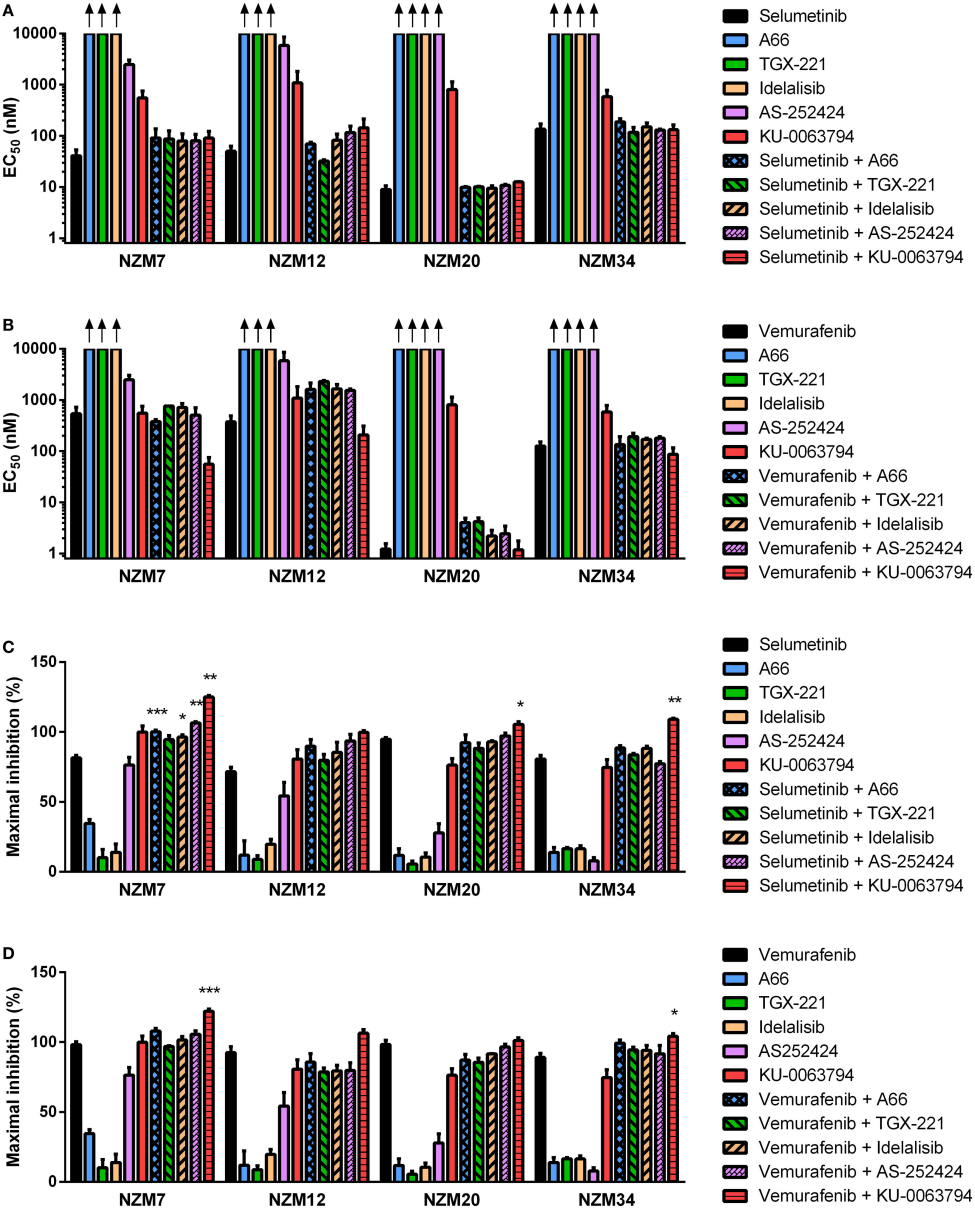
**Selumetinib and vemurafenib can interact with inhibitors of individual PI3K isoforms or mTOR to cause minor changes in potency or maximal inhibition of cell proliferation**. EC_50_ values for selumetinib **(A)** or vemurafenib **(B)** and PI3K isoform or mTOR inhibitors alone and in combination. **(C)** Maximum inhibition of cell proliferation relative to pretreatment after 72 h exposure to selumetinib or **(D)** vemurafenib and PI3K isoform or mTOR inhibitors alone and in combination at concentrations up to 10 μM. Bars represent the mean ± SEM of two to six independent evaluations. Arrows indicate EC_50_ values exceeded 10 μM. Statistical significance of differences between mean values was evaluated by one-way ANOVA with Dunnett’s multiple comparison analysis. *P < 0.05; **P < 0.01; ***P < 0.001 relative to both single agent treatments.

Since ZSTK474 and BEZ235 were able to interact synergistically at EC_50_ or induce greater reductions in maximal inhibition of cell proliferation in combination with selumetinib and vemurafenib in the cell line panel, we next investigated whether inhibitors of individual PI3K isoforms or mTOR could have a similar impact. NZM7, NZM12, NZM20, and NZM34 were treated with the p110α inhibitor A66, the p110β inhibitor TGX-221, the p110δ inhibitor idelalisib, the p110γ inhibitor AS-252424, or the mTORC1/2 selective inhibitor KU-0063794 alone or in combination with selumetinib or vemurafenib. The PI3K isoform-selective inhibitors were ineffective at inhibiting cell proliferation as single agents with EC_50_ values in excess of 10 μM for all inhibitors in all cell lines, except AS-252424 in NZM7 (EC_50_ = 2.5 ± 0.6 μM) and NZM12 (EC_50_ = 5.9 ± 2.7 μM) (Figure 7A). By contrast, KU-0063794 more potently inhibited cell proliferation with EC_50_ values ranging from 0.6 ± 0.2 to 1.1 ± 0.7 μM across the four cell lines. There was no synergistic interaction in potency when these agents were combined with selumetinib or vemurafenib, except for KU-0063794 with vemurafenib in NZM7 cells, where strong synergy was observed (CI = 0.20 ± 0.05) (Figure 7B).

Despite the lack of synergy in drug potency, significant increases in maximal growth inhibition at concentrations up to 10 μM were observed in NZM7 cells with combination therapy of selumetinib and A66, idelalisib, or AS-252424 (*P* < 0.05) and in NZM7, NZM20, and NZM34 cells with selumetinib and KU-0063794 (*P* < 0.05) (Figure 7C). However, unlike with ZSTK474 or BEZ235 (Figures 3C,F and 4C,F), the increase in maximal growth inhibition induced by A66, idelalisib, or AS-252424 in combination with selumetinib was only observed with high-drug concentrations (data not shown). No significant changes in maximal growth inhibition were observed for any of the vemurafenib and PI3K isoform inhibitor combinations, but maximal growth inhibition was significantly increased by vemurafenib and KU-0063794 relative to either agent alone in NZM7 (*P* < 0.001) and NZM34 (*P* < 0.05) cells (Figure 7D).

### Selumetinib and ZSTK474 or BEZ235 synergistically inhibit tumor growth in an NZM20 xenograft model

**Figure 8:**
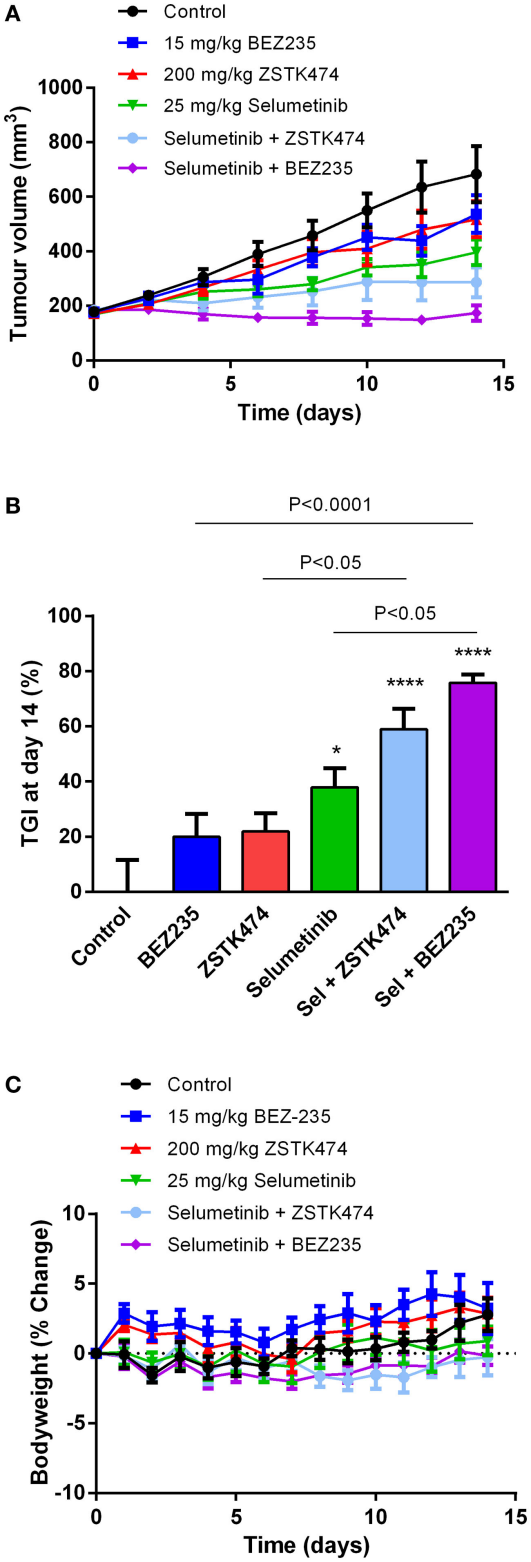
**Selumetinib (Sel) in combination with ZSTK474 or BEZ235 synergistically inhibits tumor growth in an NZM20 xenograft model**. **(A)** Average tumor volume over the 14-day dosing period. **(B)** Tumor growth inhibition (TGI) at day 14. Statistical significance of differences between mean values was evaluated by one-way ANOVA with Sidak’s multiple comparison analysis. *P < 0.05; **P < 0.01; ***P < 0.001 relative to control. Statistically significant differences between treatment groups are marked as indicated. **(C)** Bodyweight changes over the 14-day dosing period. Bars and symbols represent the mean ± SEM (*n* = 7).

To determine if selumetinib can synergize with ZSTK474 or BEZ235 to prevent tumor growth *in vivo*, NZM20 cells were inoculated into NIH-III immunodeficient mice. The NZM20 cell line was selected since it was sensitive to selumetinib (Figure 2A), showed additivity at EC_50_ and significant increases in maximal growth inhibition to ZSTK474 or BEZ235 in combination with selumetinib (Figure 3) and reproducibly forms tumors *in vivo*. Once tumors were established at 150–200 mm^3^, animals were treated with 25 mg/kg selumetinib, 200 mg/kg ZSTK474, 15 mg/kg BEZ235, 25 mg/kg selumetinib + 200 mg/kg ZSTK474, or 25 mg/kg selumetinib + 15 mg/kg BEZ235 by oral gavage daily for 14 days. These dose levels were selected as they were known to be well tolerated and to partially inhibit tumor growth or pAKT or pERK expression as single agents (36, 43, 44). ZSTK474 and BEZ235 were able to inhibit tumor growth by 21.8 ± 6.6 and 19.9 ± 8.3% relative to control, respectively, by day 14, but neither change reached statistical significance, while selumetinib significantly inhibited tumor growth by 37.9 ± 6.9% (*P* < 0.05) relative to control at day 14 (Figures 8A,B). Both combination regimens synergistically inhibited tumor growth from day 2 (BEZ235 and selumetinib) or day 4 (ZSTK474 and selumetinib) onward, with significant TGIs of 75.8 ± 3.1% (*P* < 0.0001) and 59.0 ± 7.4% (*P* < 0.0001) relative to control at day 14 for selumetinib with BEZ235 and ZSTK474, respectively. Treatment with selumetinib and BEZ235 prevented tumors from growing above baseline levels during the treatment period and at day 14 tumor size was significantly reduced from selumetinib (*P* < 0.05) or BEZ235 (*P* < 0.0001) treatment alone. All treatments were well tolerated over the dosing period, with minimal weight change seen in all groups (Figure 8C).

## Discussion

The introduction of BRAF and MEK inhibitors into clinical practice has improved outcomes for patients with metastatic *BRAF*-mutant melanoma. However, both intrinsic and acquired resistances limit the use of these agents in the clinic (2, 3, 6). Combination therapy with BRAF and MEK inhibitors has proved beneficial in overcoming ERK-dependent acquired resistance (5), but current strategies to overcome intrinsic resistance or to identify determinants of sensitivity are limited. Since signaling through the PI3K/AKT/mTOR pathway has been implicated in intrinsic resistance to BRAF or MEK inhibitor therapy, we investigated if individual or pan isoform inhibitors of PI3K and/or mTOR could enhance the antitumor activity of BRAF/MEK inhibitors in *BRAF*-mutant melanoma cell lines that are sensitive to BRAF/MEK inhibition.

A panel of nine early passage *BRAF*-mutant metastatic melanoma cell lines that were developed and maintained at 5% oxygen tension to mimic physiological oxygen levels in the tumor microenvironment was selected that displayed a range of sensitivities to selumetinib and vemurafenib. All the cell lines were considered to be sensitive to BRAF or MEK inhibition, as intrinsically resistant cell lines typically have EC_50_ values in excess of 5 μM in response to BRAF or MEK inhibitors (19, 32, 45). Cells that were most sensitive to BRAF inhibition with vemurafenib were also sensitive to MEK inhibition with selumetinib, as has previously been reported (19) with the exception of NZM43, a V600K mutant line, which was nearly 100-fold more sensitive to MEK than BRAF inhibition. The cell lines that were most sensitive to BRAF or MEK inhibition expressed low levels of pAKT and EGFR and were wild type for *PTEN*. These results were largely expected since loss of functional PTEN (16), EGFR upregulation (46) and high basal expression of pAKT (18–20) have been implicated in resistance to BRAF and MEK inhibition.

Single agent therapy with selumetinib or vemurafenib was cytostatic in the majority of cell lines, being unable to inhibit cell proliferation to pretreatment levels, despite selumetinib achieving substantial reduction of pERK in excess of the 80% inhibition required for *in vitro* sensitivity and clinical activity (25, 47). This suggests that while ERK signaling was the main driver of growth in these cell lines, other signaling pathways also contributed. The exceptions were the highly sensitive NZM3 and NZM11 cells, where selumetinib and vemurafenib both induced a cytotoxic response on cell proliferation. Since these cells expressed low levels of pAKT, it is likely that they were highly dependent on ERK signaling and so BRAF or MEK inhibition alone was sufficient to prevent cell growth.

Combined MEK and BRAF inhibition has proven beneficial in the clinic to overcome ERK-dependent acquired resistance in *BRAF*-mutant melanoma patients (5). Here, synergy was observed in 7/9 *BRAF*-mutant cell lines when selumetinib and vemurafenib were applied in combination. However, the combination was less effective in cell lines that had low-pERK expression, where ERK signaling is less likely to play a major role in oncogenesis. Notably, maximum growth inhibition with selumetinib and vemurafenib in combination was unchanged relative to both agents alone, largely because these agents targeted the same signaling pathway in the absence of ERK-dependent acquired resistance in this short duration cell proliferation model. As a result, combination therapy with selumetinib and vemurafenib remained cytostatic in several cell lines.

Inhibition of PI3K alone with ZSTK474 or PI3K and mTOR with BEZ235 was effective at inhibiting cell proliferation and AKT signaling in all *BRAF*-mutant melanoma cell lines. PI3K inhibitors have previously shown activity in melanoma, regardless of *BRAF* mutation status (32, 48), in response to PI3K family members being highly expressed in metastatic melanoma, and *PTEN* being frequently deleted (49). However, compensatory signaling through the RAS/RAF/MEK/ERK pathway following tyrosine kinase receptor upregulation or pathway cross-talk can lead to resistance to PI3K inhibitors, particularly in *BRAF*-mutant melanoma where ERK signaling is enhanced, warranting the need for combination therapy (27–31). Synergy was observed for vemurafenib and ZSTK474 or BEZ235 combinations in several cell lines, but was less pronounced for selumetinib combinations, possibly due to the greater potency of selumetinib over vemurafenib as a single agent. However, in all cell lines, regardless of whether synergy was observed, treatment with selumetinib or vemurafenib in combination with either ZSTK474 or BEZ235 generated a cytotoxic response. Other than NZM3 and NZM11, where BRAF or MEK inhibition alone was sufficient for cytotoxicity, the remaining cell lines all benefited from dual inhibition with significantly greater maximal inhibition of proliferation with at least one of the BRAF/MEK inhibitor and PI3K or PI3K/mTOR inhibitor combinations relative to each agent on their own. These results suggest that even BRAF or MEK inhibitor sensitive *BRAF*-mutant melanoma cell lines are not entirely dependent on ERK signaling for their growth. Cells that express pAKT can still proliferate in the absence of ERK signaling and so inhibition of both ERK and AKT signaling (as achieved by selumetinib and ZSTK474 or BEZ235 combinations) is required for cytotoxic inhibition of growth.

Isoform-selective inhibitors of PI3K lacked potency at inhibiting cell proliferation on their own, but A66, idelalisib, and AS-252424 were able to significantly increase maximal inhibition when combined with selumetinib, but not vemurafenib, in NZM7 cells. However, since the isoform-selective PI3K inhibitors only enhanced selumetinib activity at concentrations approaching 10 μM, it is possible that these effects could be due to loss of isoform selectivity at these high concentrations. Nevertheless, the activity was less than that observed for ZSTK474, suggesting that although one or more of the PI3K isoforms may play a preferential role in promoting survival in *BRAF-*mutant melanoma cell lines, inhibition of all or most isoforms is required due to the ability of isoforms to readily compensate for one another (50, 51). This is consistent with our previous findings that isoform-selective PI3K inhibitors are ineffective at inhibiting pAKT expression on their own at low concentrations, except for p110α-selective inhibitors in H1047R PIK3CA mutant lines, but in combination can substantially inhibit pAKT expression (36). By contrast, KU-0063794 demonstrated strong synergy in combination with vemurafenib in NZM7 cells and increased maximal growth inhibition in NZM7, NZM20, and NZM34 cells with selumetinib or vemurafenib. By inhibiting mTORC1 and 2, KU-0063794 can prevent pS6 and pAKT expression (52) with pS6 inhibition previously having been shown to predict response to and to synergize with BRAF or MEK inhibition (25, 26, 32, 33, 48).

Substantial inhibition of pS6 was observed here with selumetinib alone in the cell lines that were most sensitive to selumetinib or vemurafenib and expressed low pAKT, while only minor inhibition was detected in less sensitive lines that expressed moderate to high levels of pAKT. Further inhibition of pS6 was achieved in combination with ZSTK474 and near complete inhibition in combination with BEZ235. These results corroborate with previous findings that mTORC1 activity is primarily regulated by ERK signaling in BRAF or MEK inhibitor sensitive melanomas (25). However, in cell lines that express pAKT, AKT is likely to become the dominant regulator of mTORC1, such that inhibition of PI3K/mTOR signaling, as achieved by BEZ235 and to a lesser extent ZSTK474, is required for substantial inhibition of pS6 (25, 26). Since BEZ235 and ZSTK474 were able to induce similar inhibition of pAKT and pERK in combination with selumetinib, the enhanced inhibition of pS6 with BEZ235 and selumetinib is likely to help explain why this combination was more effective than ZSTK474 and selumetinib at preventing tumor growth in the NZM20 xenograft model. However, as differences in the pharmacokinetic or pharmacodynamic properties of the drugs in combination could also contribute to the observed variations in efficacy *in vivo*, these properties will need to be evaluated in future alongside an assessment of optimal dose levels and scheduling of these agents in combination.

Overall, these results suggest that while some BRAF/MEK inhibitor sensitive *BRAF*-mutant melanomas, particularly those that express low pAKT and functional PTEN, can be effectively treated with single agent BRAF or MEK inhibition, for the majority of lines, enhancement of antitumor activity can be achieved by combining BRAF or MEK inhibition with an inhibitor of PI3K/mTOR signaling to block ERK, AKT, and S6 signaling. Despite either inhibition of PI3K or mTOR synergizing with BRAF/MEK inhibition, dual PI3K/mTOR inhibition appears the most promising strategy to enhance sensitivity to BRAF and MEK inhibition and possibly overcome ERK-independent intrinsic or acquired resistance.

## Author Contributions

MS, PS, and SJ conceived and designed the experiments. MS, ER, and SK performed the experimental studies. GR performed compound synthesis. BB established the melanoma cell lines. Data were analyzed by MS, SK, and SJ. SJ drafted the figures, tables, and manuscript. BB and PS critically revised the paper and all authors approved the final version.

## Conflict of Interest Statement

The research was conducted in the absence of any commercial or financial relationships that could be construed as a potential conflict of interest.
